# Data collected to measure the impact of problem-based learning and document physics classroom practices among Ugandan secondary schools

**DOI:** 10.1016/j.dib.2022.108534

**Published:** 2022-08-09

**Authors:** Stella Teddy Kanyesigye, Jean Uwamahoro, Imelda Kemeza

**Affiliations:** a African Centre of Excellence for Innovative Teaching and Learning Mathematics and Science (ACEITLMS), University of Rwanda College of Education (URCE), Kayonza, PO BOX 55, Rwamagana, Rwanda; b Mbarara University of Science and Technology, Mbarara, Uganda

**Keywords:** Data, Classroom observation, Mechanical waves, Problem-based learning, Views about science, Uganda

## Abstract

This dataset comprises data collected from three measures; (a) 419 students who completed the mechanical waves conceptual survey (MWCS), (b) the same students (419 students) who completed the views about sciences survey (VASS), and (c) 152 physics lessons that were observed from 22 teachers using reformed teaching observation protocol (RTOP). The data were collected from 19 schools in Mitoma district, Southern western Uganda, for the first author's doctoral research project in physics education pursued at the University of Rwanda College of Education (URCE). The data were collected from February to April 2021, while the training for the problem-based learning (PBL) approach was delivered to teachers from 10 to 11 February 2021. The students were split into four groups (with a Solomon four-square design), and data includes pre-and post-test measures from before and after teacher instruction on problem-based learning. On the side of teachers, 72 classrooms were observed in classrooms taught by teachers who were trained in PBL, while 80 were observed in the classroom taught by teachers who were not trained in PBL. This dataset is in the form of raw data, and it can be analyzed in various forms, such as students' performance and conceptual understanding of mechanical waves, their attitudes toward science, and teacher and students' classroom practices. It provides room for researchers to explore, dig deep into, and reuse it for various purposes such as experimental versus control trials, students' gender, school characteristics, etc. Policymakers and educationists would also explore and get insights into Ugandan classrooms' teaching and learning practices.

**Specifications Table**

**Table:** 

Subject	Physical sciences
Specific subject area	Physics education
Type of data	MS Excel spreadsheets Table
How data were acquired	The data were acquired during field visits to 19 schools. Three types of data were collected using standardized research instruments. The first one was test scores collected using mechanical waves conceptual survey (MWCS) [1]. The second one was attitude scales collected using views about sciences survey (VASS) [2]. The third one was classroom practices collected using reformed teaching observation protocol (RTOP) [3]. All these tools are available at https://www.physport.org/assessments/. The data were collected on papers in English and later recorded in MS Excel 2016.
Data format	Raw Filtered
Parameters for data collection	Prior to data collection, researchers presented an ethical clearance from the ethical committee at the University of Rwanda College of Education (URCE), located in Kayonza, Rwanda, to local and educational officials in Uganda. Teachers and students were assured anonymity and confidentiality of their identifications and information, respectively. All research instruments are valid and reliable as they are proven standard level in an international context.
Description of data collection	About 419 physics students sat for the MWCS and VASS attitude tests before and after the problem-based learning (PBL) intervention. About 152 lessons were observed during teaching and learning activities from February to April 2021. MWCS comprises 22 questions, VASS comprises 33 items, while RTOP comprises 25 statements. During classroom observation, researchers had to observe the lesson and write notes, then fill out the RTOP form after the class ended.
Data source location	Institution: 19 Secondary schools City/Town/Region: Mitoma district, South-western Country: Uganda Samples/data: 419 students and 22 physics teachers
Data accessibility	Data is freely available to explore and reuse. Repository name: Mendeley Direct URL to data: https://data.mendeley.com/datasets/rdtcgstmps/3

## Value of the Data

• These data are of great importance because they provide insights into Ugandan classrooms, students' performance of mechanical waves and their attitudes in learning sciences such as physics, and teachers' implementation of active learning technique –problem-based learning (PBL).
• Policymakers and Curriculum designers can base on this study to do relevant reviews that reflect competence skill acquisition among students, advocate for a Competence-Based Curriculum (CBC), and determine professional development needs for the teachers.
• Researchers in similar fields can follow these results to measure the effect of PBL and identify gaps and possible remedies for mechanical waves hence providing a reference point. Data can be analyzed into various variables such as experiment versus control groups and students, teacher, and school characteristics.

## 1 Data Description

We collected three sets of data [4] to measure the effect of problem-based learning (PBL) on students’ performance, conceptual understanding, and attitude and strengthen teachers’ effective instruction in Ugandan secondary schools. Each set is filed into one MS Excel 2016 file. The first set contains data related to students’ scores in mechanical waves titled “Ugandan Secondary Form 6 Responses on Mechanical Wave Conceptual Survey [Feb-Apr 2021].” The second file contains data related to students’ views about science titled “Ugandan Secondary Form 6 Students Views About Sciences Survey [Feb-Apr 2021].” The third file contains data related to classroom observation titled “Reformed teaching observation classroom practices in Ugandan Secondary Form 6 [Feb-Apr 2021].”

### 1.1 Data related to mechanical waves conceptual survey and views about science survey

**Fig. 1:**
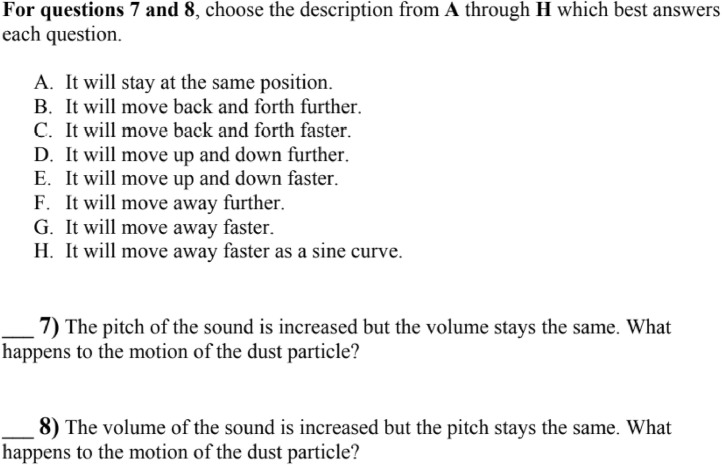
Sample of MWCS items.

The files containing the above-mentioned data are quite similar. Each file has two sheets, where the first sheet contains raw data of pre-and post-test scores and the second sheet contains an explanation of variable codes. MWCS contains 22 questions, but since five of 22 questions have extended explanatory items, MWCS contains 27 items. The development of the test is available in Barniol and Zavala’s study [5], while all items and answer keys (scoring) are available at https://www.physport.org/assessments/assessment.cfm?I=159&A=MWCS2. Above the data, correct answers are mentioned so that one can identify or count the correct among answer choices. In the data, letters were replaced by numbers for easy counting. For instance, one was used for A, 2 for B, etc. Fig. 1 shows an example of an MWCS item and possible answer choices.

**Fig. 2:**
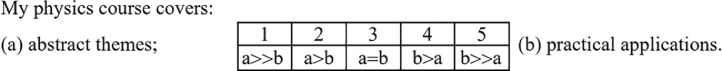
Example of VASS type of item and its rating.

Contrariwise, VASS made up of 33 items, does not have any correct answer; thus, items are rated on a 5-point scale, and each scale an ‘a’ revealing a negative attitude and a ‘b’ statement revealing a positive attitude (see Fig. 2). The first scale (a) >> (b) means mostly (a), rarely (b); the second scale (a) > (b) means more (a) than (b); the third scale (a) = (b) means equally (a) and (b); the fourth scale (b) > (a) means more (b) than (a); and the fifth scale (b) >> (a) means mostly (b), rarely (a). The development of the survey is available in Halloun and Hestenes’s study [6] and at https://www.physport.org/assessments/assessment.cfm?I=43&A=VASS.

In each file, students and school codes are made to show the reader trends of data. After these codes, eight variables follow and show the origin of pre-test and post-test scores. These variables are experimental versus control group, given both pre-test and post-test versus given only post-test, student gender, student age, subject combination, the status of the school, category of the school, and school ownership. The repeated code of the teacher shows how many times he/she was observed. Each variable was assigned a code number (see Table 1).

**Table 1:** Variables and assigned codes in MWCS and VASS.

Variable	Code number
Experimental vs control group	1 = experimental group; 2 = control group

Given both pretest and posttest vs given only posttest	1 = given both pretest and posttest; 2 = given only posttest

Gender	1 = female; 2 = male

Age	1 = 16 years and below; 2 = 17 years; 3 = 18 years; 4 = 19 years, 5 = 20 years and above

Subject combination (study option at high school)	0 = Not indicated; 1 = PEM (PHYSICS, ECONOMICS, MATHEMATICS); 2 = PCB (PHYSICS, BIOLOGY, CHEMISTRY); 3 = PCM (PHYSICS CHEMISTRY, MATHEMATICS); 4 = PENTM (PHYSICS, ENTREPRENEURSHIP, MATHEMATICS); 5 = PAM (PHYSICS, AGRICULTURE, MATHEMATICS)

Status of the school	1 = Single girls; 2 = Mixed

Category of the school	1 = Boarding only; 2 = Day only; 3 = Both day and boarding

School ownership	1 = Government; 2 = Private

By “subject combination,” this means “study option” at high school. After general education, students choose to continue at high school with a specific stud option. Thus, they combine three major subjects for science students such as PHYSICS, ECONOMICS, and MATHEMATICS (PEM), PHYSICS, BIOLOGY, and CHEMISTRY (PCB), PHYSICS CHEMISTRY, and MATHEMATICS (PCM), PHYSICS, ENTREPRENEURSHIP, and MATHEMATICS (PENTM), PHYSICS, AGRICULTURE, and MATHEMATICS (PAM).

### 1.2 Data related to reformed teaching observation protocol

The file for classroom observation data also contains two sheets—the first sheet with raw data and the second with an explanation of RTOP scales. The sheet for data display background information (variables of analysis). After the code of the observed lesson, the school code and the teacher’s code are presented in Table 2.

**Table 2:** Variables and assigned codes in RTOP.

Code name	Code number
Category of the school	(1 = Boarding only; 2 = Day only; 3 = Both day and boarding)
School ownership	(1 = Government; 2 = Private)
Students did pre-test or not	(1 = both pre-test and post-test 2 = No pre-test but only post-test)
Gender of the teacher	(1 = Male; 2 = Female)
Announced observation	(1 = Yes or 2 = No)
Years of teaching experience	(1 = Less than 5 years; 2 = 5 = 10 years; 3 = Above 10 years)
Teaching certificate	(1 = Diploma; 2 = Bachelors; 3 = Postgraduate
Teacher trained in PBL or not	(1 = Teacher trained in PBL; 2 = teacher not trained in PBL

Other information includes Subtopics observed, Date of observation, Start time and End time, Space, and Seating arrangement. One hundred and fifty two class sessions (75 in PBL and 80 in non-PBL classroom) were observed from 22 teachers. Observed classes from classes where students did pre-test or not (under Solomon design can also be identified through students’ tests and teacher training codes. RTOP has two parts; (i) background and (ii) contextual background and activities. The contextual background and activities comprise 25 items divided into three themes. These themes are lesson design and implementation (from item1 to item5), content (from item6 to item55), and classroom culture (from item16 to item25). The content theme has two sub-themes, Propositional knowledge (from item6 to item10) and Procedural Knowledge (from item11 to item15). At the same time, classroom culture has two subthemes too, Communicative Interactions (from item16 to item20) and Student/Teacher Relationships (from item21 to item25). Thus, all these themes and subthemes are disposed above the data. The data are in numbers, from 0 to 4, where 0: the behavior never occurred, 1: the behavior occurred at least once, 2: the behavior occurred more than once; very loosely describes the lesson, 3: a frequent behavior or fairly descriptive of the lesson, and 4: pervasive or extremely descriptive of the lesson. The development of RTOP is available in Pibun and Sawada's study [7] and at https://www.physport.org/assessments/assessment.cfm?I=50&A=RTOP.

## 2 Experimental Design, Materials and Methods

This dataset is aimed to measure the impact of PBL instruction among Ugandan students and document classroom practices among physics teachers and students. A Solomon four-group design [8,9] was employed during teaching and learning mechanical waves. A total of 419 physics students from 19 schools were randomly selected in Mitoma district, Southern western Uganda. Prior to random sampling, day schools alone and boarding schools alone were excluded. Thus, we only used the schools that host both day and boarding students. We included both private and public schools and both single girls and mixed schools using cluster sampling. All sixth-grade physics students were used as a whole class in these schools. Thus, the grade was purposively selected based on the unit of learning (mechanical waves). The assignment of schools to experimental and comparison (control) groups was also based on simple random sampling. Experimental allocation followed Solomon's randomized four-group design. The experimental group that performed both Pre-and Post-test accommodated 132 students, a control group that performed both Pre-and Post-test accommodated 107 students, while 99 students were assigned to the experimental group that performed only Post-test, and 81 students were assigned to a control group that performed only Post-test.

The data were collected from February 2021 to April 2021. Thus, the whole teaching intervention took three months. The pre-test for MWCS and VASS was administered in the first week of February, while the corresponding post-test was administered at the end of April 2021. RTOP was implemented throughout this period (among teachers who got PBL training and those who did not receive it). The main objective of training teachers was to provide background information on the origin and importance of PBL, skills in generating PBL questions, skills in the presentation of a PBL lesson, and knowledge on the assessment of a PBL lesson. The physics teachers at the selected schools first took a 2-day specialized training course at PBL, facilitated by the researcher (first author). At the end of the training, participants were able to write questions about the concept of electromagnetic waves using online resources and textbooks. On the first day, the origin of PBL and the importance of PBL in teaching and learning were presented by the researcher using PowerPoint slides. After that, an open discussion occurred between the researcher and teachers. The same day, teachers were given the opportunity to formulate a PBL question. They then implemented the formulated PBL questions in-class activity in terms of microteaching. The researcher presented the steps followed in presenting a PBL lesson on the second day. Then, teachers were allowed to draft class-activity PBL lessons. They worked in a group of four to five teachers and presented the formulated PBL-based class activities using flip charts. The same day, the researcher presented how to assess a PBL lesson, and then we ended with an open discussion.

### 2.1 Methods for Mechanical Waves Conceptual Survey (MWCS) data

The first data was test scores collected using mechanical waves conceptual survey (MWCS) originally developed by Apisit Tongchai and colleagues from Thailand and Australia [1] in 2008 and revised by Barniol and Zavala [5] in 2016. MWCS is in the form of pre/post-multiple-choice questions that takes 30 min to complete. It focuses on wave and optics content knowledge (mechanical waves, wave propagation, wave superposition, reflection, and standing waves). It covers intermediate, Intro-college, and high school levels [10]. The data were collected on paper and later recorded in MS Excel 2016. The current data got a reliability coefficient of .69 Cronbach alpha of internal consistency. MWCS data provides the actual answer provided by each participant, rather than providing 0s and 1s for incorrect and correct. The data is provided this way because it is in some way useful to know which wrong answers a student provided. The analysis of MWCS data may be done by counting the total score that each student got and measuring students' performance or achievement; while counting the number of students who answered each item correctly, a conceptual understanding and alternative conceptions are revealed [11].

### 2.2 Methods for Views About Science Survey (VASS) data

The second data was attitude scales collected using views about sciences survey (VASS) developed by Ibrahim Halloun from Lebanon [2] in 1996. VASS is in the form of pre/post multiple questions that take 40 min to complete. It focuses on beliefs and attitudes (structure and validity of scientific knowledge, scientific methodology, learnability of science, reflective thinking, and personal relevance of science. It covers intro-college and high school [2]. The data were collected on paper and later recorded in MS Excel 2016. The current data got a reliability coefficient of .73 Cronbach alpha of internal consistency. The analysis of VASS is done by counting the number of students who selected a certain scale or computing the average of the scale along with each respondent (student) [12].

### 2.3 Methods for Reformed Teaching Observation Protocol (RTOP) data

**Fig. 3:**
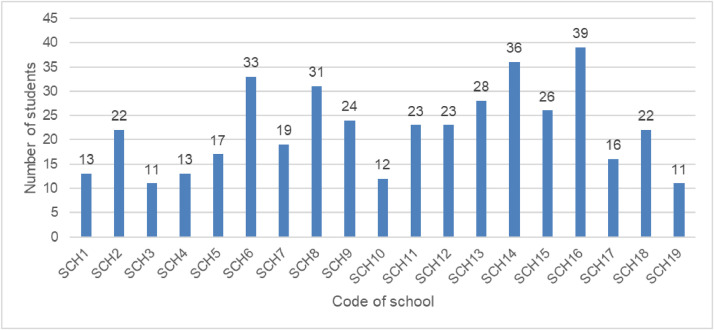
Number of students from each school.

The third data was classroom practices collected using reformed teaching observation protocol (RTOP) by Daiyo Sawada and Michael Piburn from Nothern America [3] in 2000. All these research instruments are valid and reliable and have proven their usability in international contexts [1], [2], [3]. The data were collected on paper and later recorded in MS Excel 2016. Before data collection, we checked interobserver reliability. Two observers observed one lesson and compared their ratings. The agreement of 96% and .82 Kappa statistics between two observers were attained across all 25 RTOP items. However, there was not any interrater reliability obtained throughout the course of the study itself; thus, there was no measure of observer error within the RTOP data. The observations from teachers who did not get PBL training are considered a control class, while those who received training are considered an experimental class. The workshop with teachers about learning the use of PBL instruction took place on 10th and 11th February 2021. It is important to inform readers and future users of the data about ratings of the RTOP that the rater was not blind to the group assignment (PBL or non-PBL) of the teachers since the rater was also the PBL trainer. The data were collected from 19 schools. The study involved 419 physics students (see Fig. 3) and 22 physics teachers (see RTOP file).

**Fig. 4:**
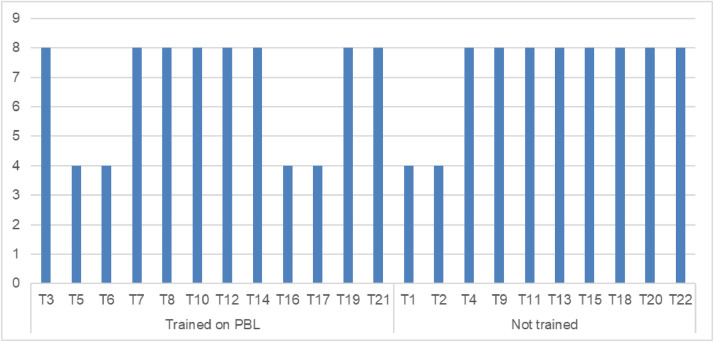
Number classroom observations from each teacher.

RTOP documents classroom practices and measure its reformed teaching [13], such as learner-centered pedagogy or active learning technique such as PBL [14]. This was used to reveal the difference PBL would make between teachers who were trained and those who were not (Fig. 4).

Teachers were observed according to their school timetable. The authors aimed to observe each teacher at least three times, as it is recommended in Ndihokubwayo et al.’s study [15]. The analysis of RTOP is done by averaging the score for each of the 25 items to see which practice is best or poorly performed by the teacher [16].

## Ethics Statement

In 2020, we submitted the research proposal to the African Center for Innovative Teaching and Learning Mathematics and Science (ACEITLMS); after the defense, we submitted it to the innovation and research unit and research at the University of Rwanda College of Education (URCE) for ethical clearance [The protocol number was 03/DRI-CE/067/EN/gi/2020]. We got ethical clearance that was used to seek permission to conduct our study. All participants have been explained the purpose of the study and all involved stages. There was no other remuneration apart from facilitation to teachers that attended our workshop on PBL. Both students and teachers agreed to participate willingly and voluntarily. They were informed that they could drop out of study any time they felt uncomfortable and were assured confidentiality in data collection.

## CRediT authorship contribution statement

**Stella Teddy Kanyesigye:** Conceptualization, Methodology, Investigation, Data curation, Writing – original draft. **Jean Uwamahoro:** Conceptualization, Validation, Data curation, Writing – review & editing, Supervision. **Imelda Kemeza:** Visualization, Validation, Writing – review & editing, Supervision.

## Declaration of Competing Interest

The authors declare that they have no known competing financial interests or personal relationships hich have or could be perceived to have influenced the work reported in this article.

## Data Availability

Data for measuring impact of problem-based learning during learning mechanical waves: MWCS, VASS, RTOP (Original data) (Mendeley Data).
